# Bibliometric analysis of chloride channel research (2004–2019)

**DOI:** 10.1080/19336950.2020.1835334

**Published:** 2020-10-24

**Authors:** Jingjing Shi, Shuqing Shi, Guozhen Yuan, QiuLei Jia, Shuai Shi, Xueping Zhu, Yan Zhou, Ting Chen, Yuanhui Hu

**Affiliations:** a China Academy of Chinese Medical Sciences, Guanganmen Hospital, Beijing, China; b Graduate School, Beijing University of Chinese Medicine, Beijing, China

**Keywords:** Chloride channel, cystic fibrosis, bibliometric, CiteSpace, visual analysis

In recent years, both cation [1]and anion channels [2,3] have emerged as significant molecules with aberrant expression, activity, and localization in various pathological conditions such as cardiovascular dysfunction, neurological disorders, metabolic diseases, and cancers. The main class of anion channels associated with multiple pathological disorders are chloride channels. Chloride channels are widely present in the cell membranes and organelle membranes of organisms. The chloride channels can transport not only Cl^−^ but also I^−^, Br^−^, F^−^, NO_3_^−^, PO_4_^3-^ and even negatively charged amino acids, so some people called it anion channel.

All the known chloride channels can be classified as members of the voltage-sensitive CLC subfamily, ligand-gated chloride channels such asγ-aminobutyric acid (GABA), and glycine receptors, calcium-activated chloride channels such as TMEM16A [4], high conductance chloride channels, the cystic fibrosis transmembrane conductance regulator (CFTR), and volume-regulated channels [5].

The opening of the chloride channel is related to membrane voltage, intracellular ATP hydrolysis, cell expansion, intracellular H^+^, Ca^2+^ concentration, intracellular residue phosphorylation, and cell signaling molecule binding.

Chloride channels are involved in a wide range of biological functions, including stabilization of cell membrane potential, maintenance of intracellular pH, cell proliferation, fluid secretion, regulation of cell volume, and acidification of intracellular organelles [5].

Mutations in several chloride channels cause human diseases, including cystic fibrosis, macular degeneration, myotonia, kidney stones, renal salt wasting, and hyperekplexia. Chloride channel modulators have potential applications in the treatment of some of these disorders, as well as in secretory diarrheas, polycystic kidney disease, osteoporosis, and hypertension.

A bibliometric study can calculate the productivity of institutions, countries, authors, and the frequency of keywords to explore research hotspots/frontiers in specific fields [6,7]. Through bibliometric analysis, researchers can summarize the current situation and development trends of research fields or specific diseases, and provide directions and ideas for future research [8]. CiteSpace and VOSviewer are the commonly used bibliometric visualization tools for data analysis and visualization [9,10].

Although chloride channels have been a hotspot of multidisciplinary research for decades, no bibliometric studies regarding the trends in chloride channel research activity have been published. Here, we collected scientific publications on chloride channel research in the past 16 years, using bibliometrics and visual analysis to explore the hotspots and frontier directions of chloride channel research and hope to provide researchers with some useful guidance.

The data search was conducted on 25 June 2020. The retrieved data were collected within one day to avoid any potential deviation due to the daily updating of the database. The search keywords entered into the database were as follows: TS = (chloride channel* OR chloride ion channel* OR Clˉ channel* OR CFTR) and language: (English). The data for analysis were retrieved from the Science Citation Index Expanded (SCI-expanded) of Web of Science Core Collection (WoSCC) database from 2004 to 2019.

In this study, the data were downloaded directly from the database as secondary data without further animal experiments. Therefore, no ethical approval was required.

**Figure 1.**
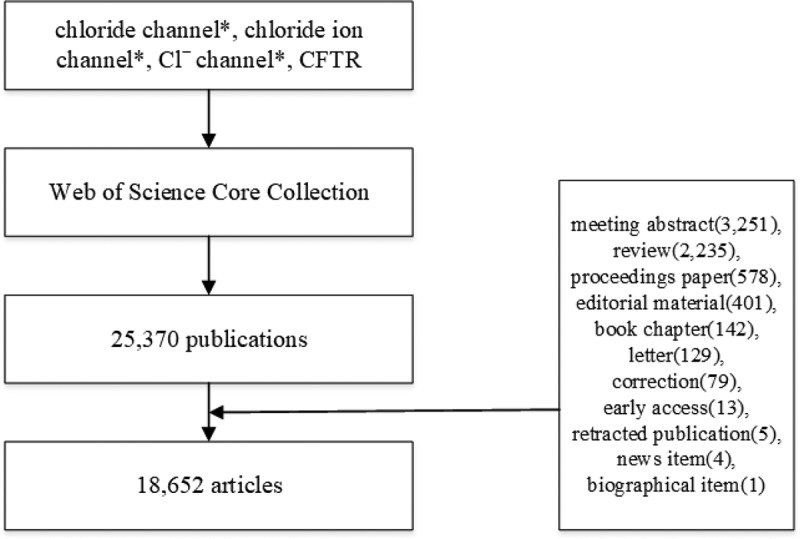
Flow chart of chloride channel researches inclusion

Twenty-five thousand three hundred and seventy publications were obtained, and the following documents were excluded: meeting abstract (3,251), review (2,235), (proceedings) paper (578), editorial material (401), book chapter (142), letter (129), correction (79), early access (13), retracted publication (5), news item (4), biographical item (1). Eighteen thousand six hundred fifty-two articles were analyzed. The retrieval strategy of the experiments is shown in Figure 1. We used the VOSviewer 1.6.11 to identify top countries, institutions, authors, and journals. The CiteSpace 5.6 R4 was used to analyze keywords, co-cited references, and trends.

**Figure 2.**
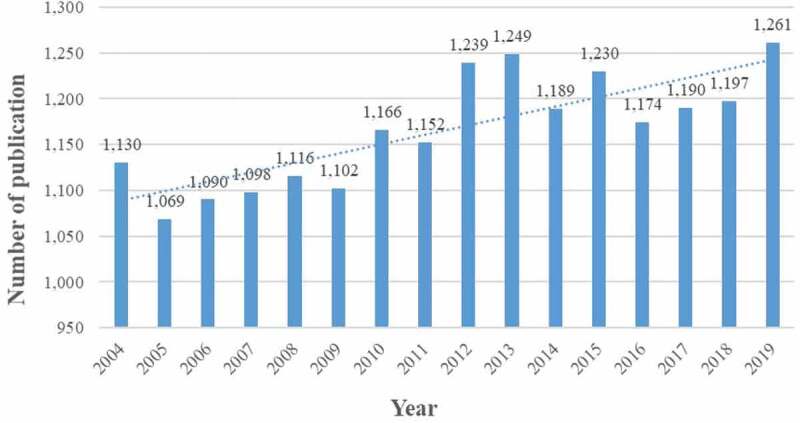
The number of annual publications on chloride channel research from 2004 to 2019

Eighteen thousand six hundred and fifty-two articles about chloride channel were published from 2004 to 2019. To explore the trends of chloride channel research, we showed the number of articles per year in the form of a histogram. As shown in Figure 2, there was an increasing trend for the number of research publications on the chloride channel, with the average annual number of publications being 1,166. The number of published articles on the chloride channel steadily increased from 2005 through 2011, and then the number of publications increased dramatically from 2012 onwards. The annual number of articles published in 2012, 2013, and 2015 was more than 1,200, which was the rapid development period of chloride channel research. In 2019, the activity in chloride channel research reached a peak.

**Figure 3.**
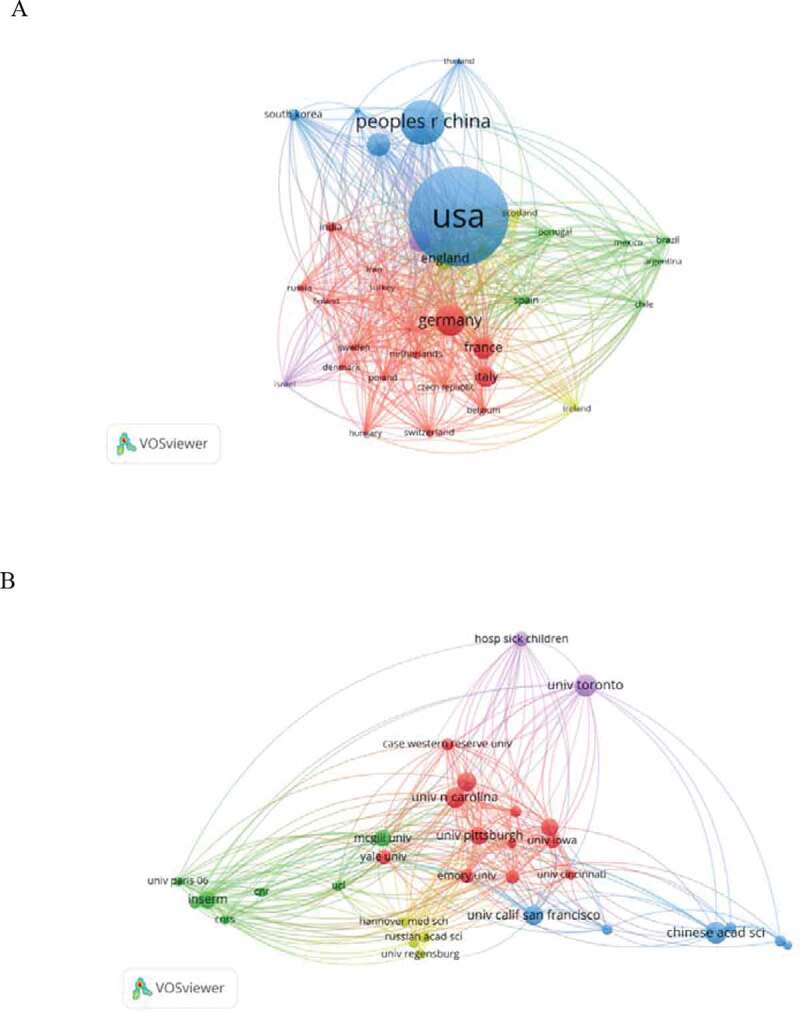
The analysis of countries and institutions. (a). Network of countries/territories engaged in chloride channel research; (b).Network of institutions engaged in chloride channel research

Co-occurrence map provides valuable information and helps researchers to identify the cooperative relationship [11]. Table 1 lists the top 10 countries and institutions contributed to publications on the chloride channel. Countries and institutions co-occurrence maps are shown in Figure 3(a,b).

**Table 1.** The top 10 countries and institutions contributed to publications on chloride channel research

Rank	Country/Territory	Frequency	Institution	Frequency
1	USA	6,688	University of Toronto	283
2	Peoples R China	2,683	Chinese Academy of Sciences	279
3	Germany	1,764	University of California	261
4	Japan	1,274	University of California, San Francisco	249
5	England	1,183	Johns Hopkins University	240
6	Canada	1,181	University of Pittsburgh	230
7	France	1,170	University of Alabama, Birmingham	226
8	Italy	1,086	Institut National de la Sante et de la Recherche Medicale (INSERM)	224
9	South Korea	603	McGill University	217
10	Australia	593	The Hospital for Sick Children	191

Researchers from more than 125 countries/territories contributed to the 18,652 articles on chloride channel research. The USA, Peoples R China, Germany, Japan, and England were the top five productive countries (Table 1). The United States published the most papers (6,688 articles), followed by China (2,683 articles), and they were the two critical countries in chloride channel research. Figure 3(a.b) shows that American institutions published most of the publications. The University of California (University of California, San Francisco) produced the highest number of publications on chloride channels (510), followed by University of Toronto (283) and Chinese Academy of Sciences (279).

The 18,652 articles were published in 2,755 journals. Table 2 lists the top 10 journals that published articles on chloride channel research. The journal of biological chemistry had the highest number at 549 (2.943%) (IF_2019_ = 4.328), followed by Plos One published 460 papers (2.466%) (IF_2019_ = 2.74 and the American journal of physiology.cell physiology ranked third at 266 articles (1.426%) (IF_2019_ = 3.485).

**Table 2.** The top 10 journals that published articles on chloride channel research

Rank	Journal	Frequency (%) N = 18,652	IF 2019	Country Affiliation
1	The journal of biological chemistry	549(2.943%)	4.238	United State
2	PLoS One	460(2.466%)	2.74	United State
3	American journal of physiology.Cell physiology	266(1.426%)	3.485	United State
4	Journal of cystic fibrosis	258(1.383%)	4.759	Netherlands
5	Journal of Physiology-London	245(1.314%)	4.547	England
6	Proceedings of the National Academy of Sciences of the United States of America	223(1.196%)	9.412	United State
7	American journal of physiology. Renal physiology.	217(1.163%)	3.144	United States
8	Scientific reports.	211(1.131%)	3.998	England
9	The journal of physical chemistry. A	201(1.078%)	2.60	United States
10	Pflügers Archiv: European journal of physiology.	193(1.035%)	3.158	Germany

Author co-occurrence map can provide information on influential research groups and potential collaborators. It can help researchers to find potential collaborators [12].

**Figure 4.**
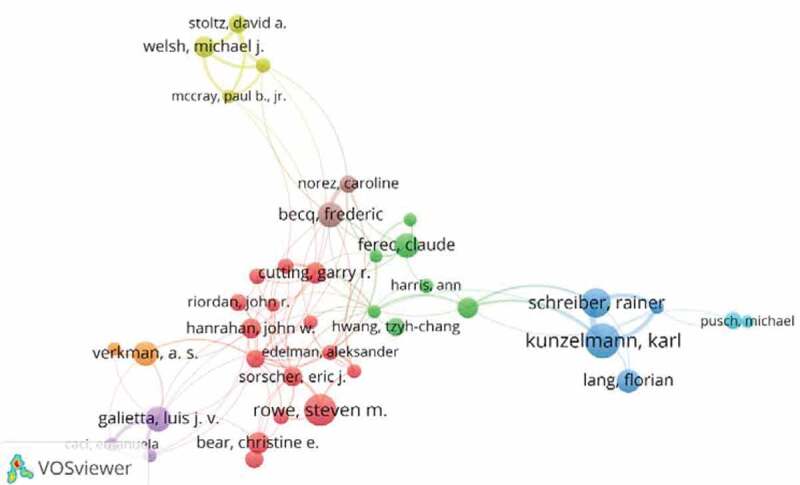
The network of authors contributed to chloride channel research

More than 70,000 authors contributed 18,652 articles related to chloride channel research. Figure 4 shows the network of authors contributed to chloride channel research, and the top10 active authors are listed in Table 3. In the network of authors contributed to chloride channel research, the largest node was Kunzelmann, Karl (82 articles) who mainly focused on TMEM16A and its role in disease [13,14]. Rowe, Steven M. was the second highly published author. His research focused on the mechanisms underlying the development and natural progression of the airway mucus defect in cystic fibrosis (CF) [15] and the clinical research of patients with cystic fibrosis homozygous for the F508del mutation [16].

**Table 3.** The top10 active authors in chloride channel research

Rank	Author	Freq
1	Kunzelmann, Karl	82
2	Rowe, Steven M.	75
3	Schreiber, Rainer	68
4	Galietta, Luis J. V.	59
5	Becq, Frederic	58
6	Verkman, A. S.	57
7	Ferec, Claude	57
8	Lang, Florian	55
9	Bear, Christine E.	51
10	Welsh, Michael J.	49
11	Cutting, Garry R.	49

**Figure 5.**
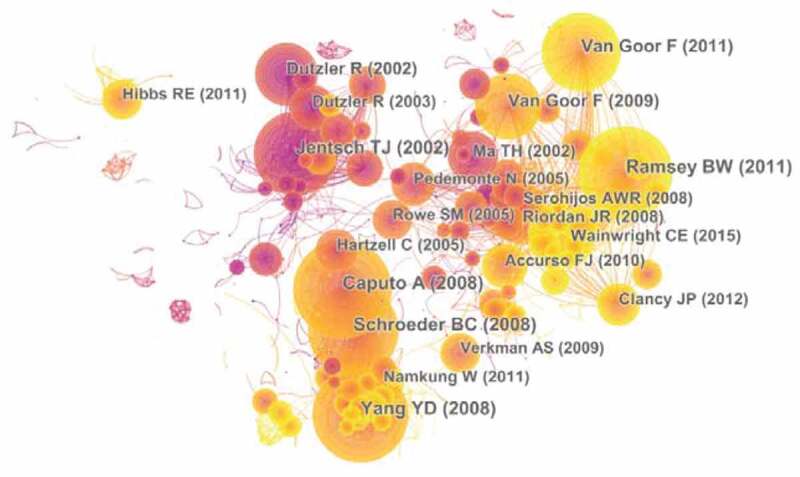
The analysis of Co-cited references: Co-citation network of references from publications on chloride channel research

Eighteen thousand six hundred and fifty-two articles were visualized and analyzed using CiteSpace with a time span from 2004 to 2019, and a time slice of 1 was chosen for the analysis of the co-cited references. The network of co-cited references on chloride channels consists of references with higher centrality and citation counts which is presented in Figure 5. The highly cited references were analyzed to determine the key knowledge base in the field. The top 10 highest co-cited references are summarized in Table 4. Caputo A, Yang YD, and Schroeder BC mainly focused on TMEM16A, which is a membrane protein associated with calcium-dependent chloride channel activity [17–19]. The highly co-cited references on TMEM16A were mainly published in 2008. In 2002, Ma TH found that thiazolidinone CFTR inhibitors may be useful in developing large-animal models of cystic fibrosis and in reducing intestinal fluid loss in cholera and other secretory diarrheas [20]. Van Goor F mainly focuses on the treatment of cystic fibrosis. In 2009, his research showed that VX-770 could restore CFTR function and rescue epithelial cell function in the human CF airway [21]. Two years later, he discovered that VX-809 represents a type of CFTR corrector, explicitly solving the underlying processing defects in F508del-CFTR [22]. In 2011, Ramsey BW indicated that ivacaftor (VX-770), a CFTR potentiator, was associated with improvements in lung function in subjects with cystic fibrosis [23]. In 2015, Wainwright, CE showed that lumacaftor in combination with ivacaftor provided a benefit for patients with cystic fibrosis homozygous for the Phe508del CFTR mutation [24]. The publications on the molecular structure and physiological functions of chloride channels laid the foundation for the in-depth study of chloride channels in 2002 [25,26].

**Table 4.** The top10 Co-cited references (CR) in chloride channel research

Rank	Freq	Author	Year	Source	Co-cited Reference
1	366	Caputo A	2008	Science	TMEM16A, a membrane protein associated with calcium-dependent chloride channel activity.
2	360	Yang YD	2008	Nature	TMEM16A confers receptor-activated calcium-dependent chloride conductance.
3	350	Ramsey BW	2011	The New England journal of medicine.	A CFTR potentiator in patients with cystic fibrosis and the G551D mutation.
4	342	Schroeder BC	2008	Cell	Expression cloning of TMEM16A as a calcium-activated chloride channel subunit.
5	315	Jentsch TJ	2002	Physiological reviews	Molecular structure and physiological function of chloride channels.
6	299	Van Goor F	2011	Proceedings of the National Academy of Sciences of the United States of America.	Correction of the F508del-CFTR protein processing defect in vitro by the investigational drug VX-809.
7	254	Van Goor F	2009	Proceedings of the National Academy of Sciences of the United States of America.	Rescue of CF airway epithelial cell function in vitro by a CFTR potentiator, VX-770.
8	237	Dutzler R	2002	Nature	X-ray structure of a ClC chloride channel at 3.0 A reveals the molecular basis of anion selectivity.
9	192	Ma TH	2002	The Journal of clinical investigation.	Thiazolidinone CFTR inhibitor identified by high-throughput screening blocks cholera toxin-induced intestinal fluid secretion
10	179	Wainwright CE	2015	The New England journal of medicine.	Lumacaftor-Ivacaftor in Patients with Cystic Fibrosis Homozygous for Phe508del CFTR.

**Figure 6.**
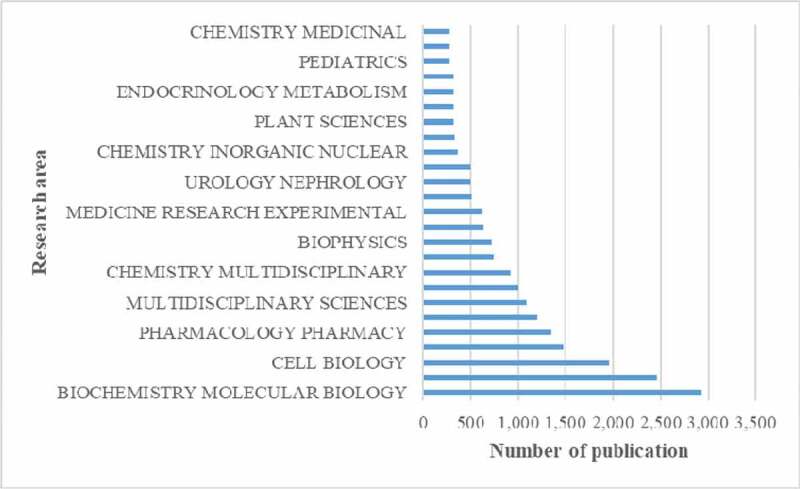
The 15 research areas on chloride channel research

Figure 6 shows the top 15 research areas that appeared in publications related to chloride channel research from 2004 to 2019. Biochemistry and molecular biology, cell biology, pharmacology pharmacy are the three areas where chloride channels are more studied.

Keywords represent the main content of research. Keyword co-occurrence analysis provides a reasonable description of research hotspots, and burst keywords can represent research frontiers over a period of time [27].

**Figure 7.**
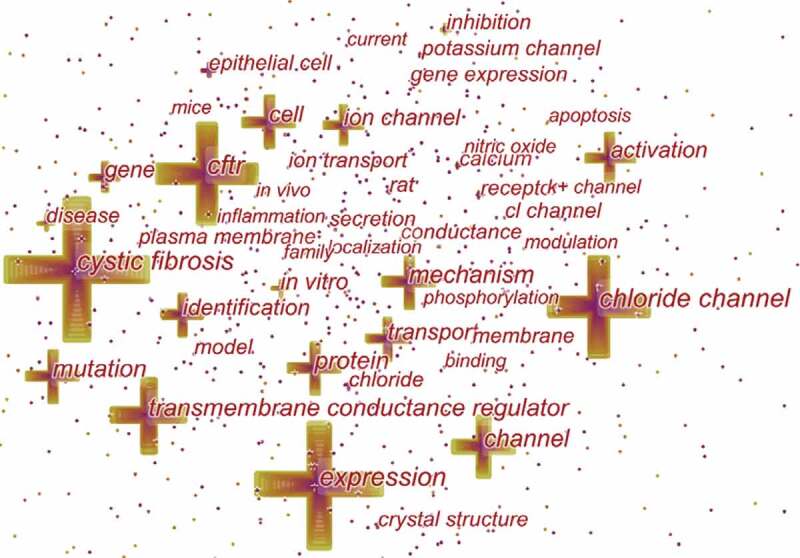
The analysis of keywords in chloride channel research

CiteSpace 5.6.R4 were used to construct acknowledge map of keyword co-occurrence (Figure 7) and identified the top 20 keywords in chloride channel research articles from 2004 to 2019 (Table 5), according to frequency. The top keywords were “cystic fibrosis,” “expression,” “transmembrane conductance regulator,” “mutation,” “mechanism,” “protein,” “activation,” “cell,” “identification,” “Transport,” “gene,” “in vitro,” “inhibition,” “disease,” “calcium,” “receptor,” “in vitro,” “epithelial cell,” “membrane,” “crystal structure,” “potassium channel.” Therefore, research hotspots can be summarized in the following aspects:

**Table 5.** Top 20 keywords in terms of frequency in chloride channel research

Rank	Keyword	Frequency	Rank	Keyword	Frequency
1	cystic fibrosis	2498	11	gene	840
2	expression	2122	12	in vitro	674
3	transmembrane conductance regulator	1519	13	inhibition	649
4	Mutation	1190	14	disease	649
5	mechanism	1152	15	calcium	623
6	protein	1146	16	receptor	616
7	activation	1084	17	epithelial cell	605
8	cell	1020	18	membrane	578
9	Identification	897	19	crystal structure	559
10	Transport	894	20	potassium channel	512

1. Cystic fibrosis

Cystic fibrosis (CF), a genetic disorder observed in people of all races and ethnicities, affects approximately 80,000 people worldwide. In 1989, the discovery that mutations cause CF in the cystic fibrosis transmembrane conductance regulator (CFTR) gene, which leads to abnormal ion transport in mucous membranes throughout the body. Further affect the function of the respiratory, gastrointestinal, and reproductive tracts [28,29].

2. Identification of cystic fibrosis transmembrane conductance regulator (CFTR) modulator

Cystic Fibrosis Transmembrane Conductance Regulator (CFTR) modulators are a class of small molecule drugs that improve the activity of the defective CFTR protein in people with cystic fibrosis, resulting in improved pulmonary function, reduction of pulmonary exacerbations, and improved nutrition. Ivacaftor, a CFTR potentiator, was the first modulator to be approved by the United States FDA in 2012 and is currently available to people with CF and responsive mutations who are at least 6 months old. When ivacaftor is combined with CFTR correctors, it improves the function of the most common CFTR mutation, F508del. Combination lumacaftor/ivacaftor and tezacaftor/ivacaftor were approved for patients homozygous for F508del in 2015 and 2018, respectively. For people homozygous for F508del, the improvement in pulmonary function is modest, so these combinations are not considered highly effective modulators. Both are ineffective for F508del heterozygotes who have a second minimal function mutation. In October 2019, the triple combination elexacaftor/tezacaftor/ivacaftor was approved by the US FDA, providing a highly effective modulator for people with CF who are homozygous or heterozygous for F508del [30].

3. Calcium-activated chloride channel

TMEM16A, a protein encoded by the gene ANO1, is a calcium-activated chloride channel robustly expressed not only in epithelial cells but also in smooth muscle cells of airways, pulmonary, and systemic vessels, gastrointestinal smooth muscle cells, and the endothelial cells of pulmonary arteries [31]. TMEM16A dysfunction is implicated in many diseases such as cancer, hypertension, and cystic fibrosis [32]. Recent research shows that enhancing the activity of TMEM16A increases epithelial fluid secretion and enhances mucus clearance independent of CFTR function. TMEM16A potentiation is a novel approach for the treatment of patients with CF and non-CF muco-obstructive diseases [33].

Keywords were identified and analyzed using strong citation bursts (Table 6) to explore the frontiers of research. In Table 6, the red line indicates the time period during which the burst keyword appears [34]. As shown in Table 6, the keywords that had strong bursts after 2017 were “g551d mutation,” “phe508del cftr,” “cystic fibrosis patient,” “voltage,” “insecticide,” ‘vx 770ʹ, “guinea pig,” “atp binding,” ‘atp release’ and “insecticide resistance.” The top four research frontiers of calcium channel research were as follows:

1. The CFTR mutation locus

**Table 6.** Top 23 keywords with the strongest citation bursts

Keywords	Year	Strength	Begin	End	2015–2019
na cl cotransporter	2015	4.9641	**2015**	2016	▃▃▂▂▂
pharmacology	2015	3.8696	**2015**	2016	▃▃▂▂▂
airway epithelial cell	2015	3.8325	**2015**	2016	▃▃▂▂▂
wild type	2015	3.7952	**2015**	2016	▃▃▂▂▂
small intestine	2015	3.7952	**2015**	2016	▃▃▂▂▂
diarrhea	2015	3.7952	**2015**	2016	▃▃▂▂▂
vas deferen	2015	5.8838	**2016**	2017	▂▃▃▂▂
airway epithelia	2015	4.6441	**2016**	2017	▂▃▃▂▂
regulated anion channel	2015	4.3343	**2016**	2017	▂▃▃▂▂
translocation	2015	4.0244	**2016**	2017	▂▃▃▂▂
plant	2015	4.0244	**2016**	2017	▂▃▃▂▂
clinical trial	2015	3.7147	**2016**	2017	▂▃▃▂▂
g551d mutation	2015	6.0602	**2017**	2019	▂▂▃▃▃
phe508del cftr	2015	6.0114	**2017**	2019	▂▂▃▃▃
cystic fibrosis patient	2015	5.1089	**2017**	2019	▂▂▃▃▃
network	2015	4.5073	**2017**	2019	▂▂▃▃▃
voltage	2015	4.2066	**2017**	2019	▂▂▃▃▃
insecticide	2015	4.2066	**2017**	2019	▂▂▃▃▃
vx 770	2015	3.9059	**2017**	2019	▂▂▃▃▃
guinea pig	2015	3.9059	**2017**	2019	▂▂▃▃▃
atp binding	2015	3.8607	**2017**	2019	▂▂▃▃▃
atp release	2015	3.6053	**2017**	2019	▂▂▃▃▃
insecticide resistance	2015	3.6053	**2017**	2019	▂▂▃▃▃

CFTR is the first single-gene disease gene discovered nearly 30 years ago [35]. CFTR is an ATP-gated, cAMP-dependent chloride channel. The basic biophysical and pathological functions of CFTR are related with the secretion of chloride ion in epithelial cells and tissues. Mutations in CFTR cause cystic fibrosis. Investigation and research found that more than 2,000 mutations have been found in the human CFTR gene, of which more than 300 are pathogenic [36], approximately 70% of all CF patients are caused by the deletion of the F508 locus, the other two most common mutations are the G542X mutation and the G551D mutation.

1. The clinical trial of VX-770 in the treatment of cystic fibrosis

VX-770 is an investigational, orally bioavailable CFTR potentiator. In 2009, Van Goor F showed that VX-700 increased the activity of wild-type and defective cell surface CFTR protein in vitro. In 2012, a study evaluated the safety and adverse-event profile of VX-770 in patients with cystic fibrosis and the G551D-CFTR mutation. The results showed that VX-770 was associated with within-subject improvements in CFTR and lung function [37]. The small molecule compound drug VX-770 is now only useful for patients with the G551D mutation, and its therapeutic effect needs further study.

1. Insecticides that act on GABA receptors

There are four main insect nervous system targets of known insecticides: acetylcholinesterase, nicotinic acetylcholine receptor, γ-aminobutyric acid (GABA), and sodium ion channels. The GABA receptor (GABA receptor chloride channel complex) is considered to be one of the most critical insecticides and nematicide targets. GABA is an inhibitory neurotransmitter released from the presynaptic terminal of the nervous system of insects and mammals [38].

Based on the WOSCC database, bibliometric and Visual analysis were used to study the characteristics of chloride channel research results from 2004 to 2019. Over the past 16 years, the number of publications on the chloride channel has been on the rise. The three hot spots of chloride channel research were “cystic fibrosis,” “identification of cystic fibrosis transmembrane conductance regulator (CFTR) modulator,” and “calcium-activated chloride channel.” The top three research frontiers were “the CFTR mutation locus,” “clinical trial of VX-770 in the treatment of cystic fibrosis,” and “insecticides that act on GABA receptors.” Bibliometric analysis of the literature on the chloride channels was important in allowing researchers to identify cooperations, find research hotspots, and predict the frontiers of chloride channel research.
